# Radiographic correlates of hallux valgus severity in older people

**DOI:** 10.1186/1757-1146-3-20

**Published:** 2010-09-16

**Authors:** Paul R D'Arcangelo, Karl B Landorf, Shannon E Munteanu, Gerard V Zammit, Hylton B Menz

**Affiliations:** 1 Department of Podiatry, Faculty of Health Sciences, La Trobe University, Bundoora, Victoria, 3086 Australia; 2 Musculoskeletal Research Centre, Faculty of Health Sciences, La Trobe University, Bundoora, Victoria, 3086 Australia

## Abstract

**Background:**

The severity of hallux valgus is easily appreciated by its clinical appearance, however x-ray measurements are also frequently used to evaluate the condition, particularly if surgery is being considered. There have been few large studies that have assessed the validity of these x-ray observations across a wide spectrum of the deformity. In addition, no studies have specifically focused on older people where the progression of the disorder has largely ceased. Therefore, this study aimed to explore relationships between relevant x-ray observations with respect to hallux valgus severity in older people.

**Methods:**

This study utilised 402 x-rays of 201 participants (74 men and 127 women) aged 65 to 94 years. All participants were graded using the Manchester Scale - a simple, validated system to grade the severity of hallux valgus - prior to radiographic assessment. A total of 19 hallux valgus-related x-ray observations were performed on each set of x-rays. These measurements were then correlated with the Manchester Scale scores.

**Results:**

Strong, positive correlations were identified between the severity of hallux valgus and the hallux abductus angle, the proximal articular set angle, the sesamoid position and congruency of the first metatarsophalangeal joint. As hallux valgus severity increased, so did the frequency of radiographic osteoarthritis of the first metatarsophalangeal joint and a round first metatarsal head. A strong linear relationship between increased relative length of the first metatarsal and increased severity of hallux valgus was also observed.

**Conclusions:**

Strong associations are evident between the clinical appearance of hallux valgus and a number of hallux valgus-related x-ray observations indicative of structural deformity and joint degeneration. As it is unlikely that metatarsal length increases as a result of hallux valgus deformity, increased length of the first metatarsal relative to the second metatarsal may be a contributing factor to the development and/or progression of hallux valgus.

## Background

Hallux valgus is a common condition affecting the forefoot in which the first metatarsophalangeal joint is progressively subluxed due to lateral deviation of the hallux and medial deviation of the first metatarsal [1,2]. The resultant deformity often leads to the development of a soft tissue and osseous prominence on the medial aspect of the first metatarsal head [3], commonly referred to as a "bunion". Hallux valgus has been reported to be highly prevalent among older people [4-6]. A recent report found that up to 37% of people over 65 years of age have some degree of the deformity [7]. The high prevalence of hallux valgus is further highlighted by the number of surgical procedures that are performed each year to correct the deformity. Coughlin and Thompson [8] estimated that there were approximately 209,000 bunionectomies performed in the US in 1991. In addition, between 1997 and 2006 there were over 46,000 first metatarsophalangeal joint surgical procedures (this includes hallux valgus and hallux limitus/rigidus) performed by private surgeons in Australia, at an approximate cost of 20 million Australian dollars [9].

The aetiology of hallux valgus is uncertain, as there are many suggested causes of the deformity, including inappropriate footwear [10], bony abnormalities (i.e. the shape of the metatarsal head [11] and the length of the first metatarsal [12]), foot pronation [13], female sex [14,15] and hereditary factors [3,15]. Two recent case-control studies found hallux valgus to be significantly associated with increasing age, female sex, pain in the knee, self-reported osteoarthritis and rheumatoid arthritis [16,17]. The resultant deformity frequently causes pain and discomfort [5] and has been identified as a risk factor for falls in older people [18]. Furthermore, three studies have found that people with hallux valgus score poorly on evaluations of health-related quality of life [17,19,20]. These findings suggest that hallux valgus does not simply cause isolated problems to the feet, but can have a broader affect on an individual.

A simple method for rating the severity of hallux valgus in the clinical setting is the Manchester Scale [21]. This rating scale incorporates four comparative photographs as a method of charting the presence and severity of hallux valgus. Clinical assessment is often supported by x-ray evaluation, with hallux valgus being considered present when the hallux abductus angle (angle formed between the longitudinal bisections of the first metatarsal and proximal phalanx) is greater than 15° on the anterior-posterior projection [22,23]. X-rays are often used to evaluate hallux valgus when surgery is being considered and to chart the success of bony realignment after surgery.

There has already been considerable investigation of the reliability of x-ray observations relating to hallux valgus [24-27]. However, it is evident that there is a lack of data identifying the relationship between the clinical appearance of hallux valgus across a wide spectrum of deformity and x-ray observations. Therefore, this study aimed to explore relationships between the clinical appearance of hallux valgus and a range of relevant hallux valgus x-ray observations in older people. Unlike previous research that has simply focused on groups either with or without hallux valgus, this study investigated hallux valgus across a broad spectrum of severity.

## Methods

This study involved performing a variety of common hallux valgus x-ray measurements and then correlating these to the clinical severity of hallux valgus using the Manchester Scale. The study utilised x-rays obtained from 201 people who were taking part in a larger study of the effect of osteoarthritis on balance and falls [28]. 205 participants were initially recruited from two sources: a retirement village and a university health sciences clinic. Invitation letters were sent to all residents of the retirement village, with a response rate of 55% (176/322). Of these, 53% (93/176) consented to having foot x-rays. Invitation letters were also sent to a randomly selected group of 1,000 patients aged over 65 years from a database of 1,128 patients attending a university health sciences clinic, with a response rate of 11.2% (112/1,000), all of whom consented to having foot x-rays. Inclusion criteria for the study involved participants being 65 years of age or older, able to walk household distances without the use of a walking aid, and normal cognition (defined as a score of >7 on the Short Portable Mental Status Questionnaire [29]). Four participants' x-rays were excluded from the analysis presented in this paper as they had previously undergone hallux valgus surgery, resulting in a total sample of 201 people (402 feet).

### Grading of hallux valgus severity

**Figure 1:**
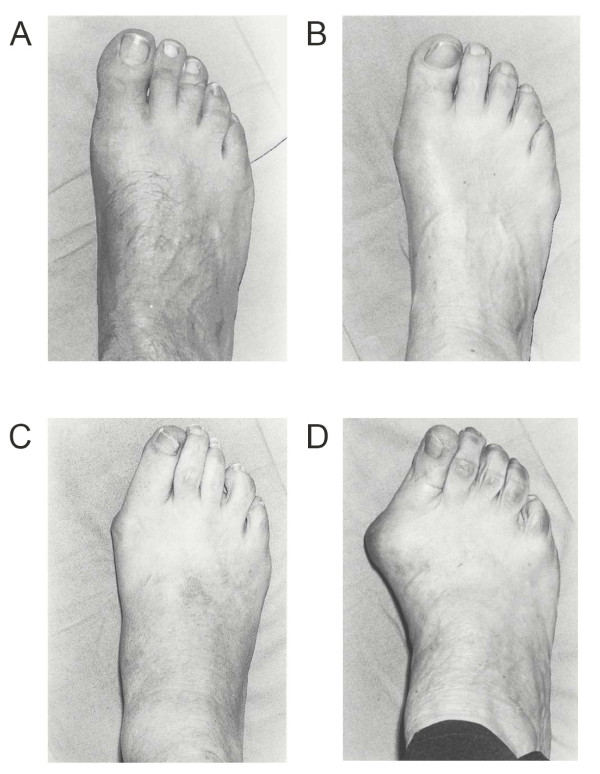
**The Manchester Scale used to determine the severity of hallux valgus (diagram adapted from Garrow et al **[21]).

The Manchester Scale, a validated tool, was applied to each foot prior to radiographic assessment, to assess the severity of hallux valgus [21,27]. This is a tool consisting of standardised photographs of feet with four grades of hallux valgus: none, mild, moderate and severe (Figure 1). Both intratester and intertester reliability of grading hallux valgus using this approach have been found to be excellent, with kappa values of 0.77 and 0.86, respectively, suggesting that it is a useful tool for clinical and research purposes [21]. Following assessment of the Manchester Scale, weight-bearing anterior-posterior and lateral radiographic projections were taken of both feet of all the participants, making a total of 402 x-rays. Measurements were obtained from the participants' left and right feet. Although we acknowledge that statistical problems can arise when assessing paired data (i.e. both feet) from an individual [30], the severity of hallux valgus is frequently asymmetrical, and in clinical practice is often assessed individually. Therefore, we considered it appropriate for the unit of analysis to be the number of feet rather than the number of participants.

### X-ray protocol

All x-ray procedures were performed according to the National Health and Medical Research Council of Australia guidelines [31]. All x-rays were carried out by the same medical imaging department using a Shimadzu UD150LRII 50 kw/30 kHz Generator and 0.6/1.2 P18DE-80S high speed x-ray tube from a ceiling suspended tube mount. AGFA MD40 CR digital phosphor plates in a 24 cm × 30 cm cassette were used. For anterior-posterior projections, the x-ray tube was angled 15 degrees cephalad and centered at the base of the third metatarsal. For lateral projections, the tube was angled 90 degrees and centered at the base of the third metatarsal. The film focus distance was set at 100 cm for both projections.

### X-ray observations

To evaluate the association between the clinical severity of hallux valgus and hallux valgus-related x-ray observations, a number of radiographic assessments were made from the hard copies of the x-ray films (Table 1).

**Table 1:** Radiographic observations.

Anterior-posterior projection	Lateral projection
Hallux abductus angle	Calcaneal inclination angle
Intermetatarsal angle	First metatarsal declination angle
Proximal articular set angle	Lateral intermetatarsal angle
Distal articular set angle	Navicular height
Sesamoid position (four grade scale)	Truncated foot length
Sesamoid position (seven position scale)	Navicular height/truncated foot length
Shape of the first metatarsal head	
Hallux abductus interphalangeal angle	
Metatarsus adductus angle	
Simplified metatarsus adductus angle	
Difference in lengths of first and second metatarsals	
Congruency of the first metatarsophalangeal joint	

Two techniques were used to bisect the first metatarsal for the measurement of the hallux abductus angle. Firstly, the traditional technique for bisecting the first metatarsal shaft was performed, where the diaphyseal region of the metatarsal is bisected. Secondly, an alternative method (as described by Miller [32]) for bisecting the first metatarsal shaft was also performed. This technique involved locating the middle of the head and the middle of the base of the metatarsal; these points were then connected via a line, which represented the bisection of the metatarsal [32]. This method has been found to be more reliable post-operatively than the more commonly used traditional method [33].

To identify if a correlation existed between the progression of hallux valgus and osteoarthritis of the first metatarsophalangeal joint, all participants were assessed for the presence of osteoarthritis. To achieve this, all radiographs were assessed using an osteoarthritis atlas developed by Menz and colleagues [34,35], whereby the presence and size of osteophytes and the extent of joint space narrowing of the first metatarsophalangeal joint are assessed. For the purpose of this study, osteoarthritis was graded as present or absent using the case definition developed by Menz and colleagues [34].

### Statistical analysis

To ensure the assessor of the radiographic observations was reliable, test-retest reliability was evaluated. To determine reliability, intraclass correlation coefficients ICC (3,1) [36], and relative (95% confidence intervals) and absolute (95% limits of agreement) reliability [37] were calculated. Intra-observer reliability consisted of the assessor measuring relevant measures on 40 randomly chosen radiographs, and then one week later re-measuring all 40 radiographs without reference to previous results. Weighted kappas were used for radiographic observations that provided ordinal data (i.e. congruency of first metatarsophalangeal joint, and the four-grade and seven-position sesamoid scales), while shape of the first metatarsal head was dichotomised into two groups ('round' and 'other') and assessed using the standard kappa statistic.

To determine the association between Manchester Scale scores (i.e. the clinical grade or severity of hallux valgus) and radiographic measurements Spearman's rho (for ordinal data) was calculated. To determine whether there were significant differences in mean radiographic measurements (e.g. angular measurements) for each of the four Manchester Scale categories, a one-way analysis of variance (ANOVA) was used for continuous data and chi-square analysis was used for categorical data. Post-hoc comparisons were performed using Bonferroni-adjusted t-tests. All data were assessed for normality prior to statistical comparisons and statistical significance was set at p < 0.05.

## Results

The 201 participants included 74 men and 127 women (402 feet), with ages ranging from 65 to 94 years. The mean age of the participants was 75.9 (±6.6) years of age. Participants reported the following medical conditions: osteoarthritis (69.7%), hypertension (59.7%), cardiac disease (20.4%), diabetes (14.9%), peripheral vascular disease (12.4%) and stroke (4%).

### Intra-tester reliability of radiographic observations

The majority of the radiographic observations were found to have very high test-retest reliability, with the majority demonstrating ICC or kappa/weighted kappa values between 0.80 to 0.99 (Table 2).

**Table 2:** Reliability of radiographic measurements (ICC = intraclass correlation coefficient, CI = confidence interval, LoA - limits of agreement).

Radiographic observations	Relative reliability ICC (95% CI)	Absolute reliability Mean difference (95% LoA)
Hallux abductus angle	0.95 (0.90 to 0.97)	-0.10 (-6.26 to 6.06)
Hallux abductus interphalangeal angle	0.66 (0.44 to 0.80)	-0.98 (-11.26 to 9.32)
Proximal articular set angle	0.90 (0.82 to 0.94)	-0.53 (-6.86 to 5.81)
Intermetatarsal angle	0.83 (0.70 to 0.90)	-0.05 (-3.43 to 3.33)
Four grade sesamoid scale	Weighted kappa = 0.86	NA
Seven position sesamoid scale	Weighted kappa = 0.90	NA
Difference in lengths of first and second metatarsals	0.89 (0.81 to 0.94)	-0.10 (-2.72 to 2.52)
Metatarsus adductus angle	0.91 (0.84 to 0.95)	-0.50 (-4.62 to 3.62)
Simplified metatarsus adductus angle	0.88 (0.79 to 0.94)	0.53 (-5.26 to 6.31)
Hallux abductus angle (Miller technique)	0.94 (0.89 to 0.97)	0.03 (-6.18 to 6.23)
Distal articular set angle	0.73 (0.54 to 0.84)	0.30 (-6.30 to 6.90)
Congruency of first MPJ	Weighted Kappa = 0.75	NA
Shape of the first metatarsal head	Kappa = 0.69	NA
First metatarsal declination angle	0.81 (0.66 to 0.89)	0.28 (-3.46 to 4.01)
Lateral intermetatarsal angle	0.90 (0.83 to 0.95)	0.03 (-2.30 to 2.35)
Calcaneal inclination angle	0.95 (0.92 to 0.97)	0.10 (-2.80 to 3.00)
Navicular height	0.89 (0.79 to 0.94)	-0.35 (-5.26 to 4.56)
Truncated foot length	0.99 (0.93 to 0.99)	-0.13 (-2.12 to 1.87)
Navicular height/truncated foot length	0.89 (0.80 to 0.94)	0.00 (-0.03 to 0.03)

### Correlations between the Manchester Scale and radiographic observations

The results revealed a strong association between the Manchester Scale scores (i.e. the clinical severity of hallux valgus) and a number of x-ray observations. Most notably, strong correlations existed between the Manchester Scale scores and the hallux abductus angle (as measured with both the traditional and Miller techniques), proximal articular set angle, sesamoid position (both techniques), and the congruency of the first metatarsophalangeal joint (Table 3). In contrast, poor correlations were noted for the metatarsus adductus angle (both the standard and simplified techniques), the distal articular set angle, and those observations relating to foot posture (calcaneal inclination angle, navicular height/truncated foot length).

**Table 3:** Associations between radiographic observations and the Manchester Scale.

Radiographic observations	Spearman's rho	p value
Hallux abductus angle	0.653	<0.001
Hallux abductus interphalangeal angle	-0.209	<0.001
Proximal articular set angle	0.554	<0.001
Intermetatarsal angle	0.426	<0.001
Four grade sesamoid scale	0.559	<0.001
Seven position sesamoid scale	0.569	<0.001
Difference in lengths of first and second metatarsals	0.313	<0.001
Metatarsus adductus angle	-0.011	0.828
Simplified metatarsus adductus angle	0.110	0.027
Hallux abductus angle (Miller technique)	0.648	<0.001
Distal articular set angle	-0.104	0.037
Congruency of first MPJ	0.586	<0.001
Shape of the first metatarsal head	0.311	<0.001
First metatarsal declination angle	-0.097	0.052
Lateral intermetatarsal angle	-0.006	0.906
Calcaneal inclination angle	-0.053	0.287
Navicular height/truncated foot length	-0.221	<0.001

**Figure 2:**
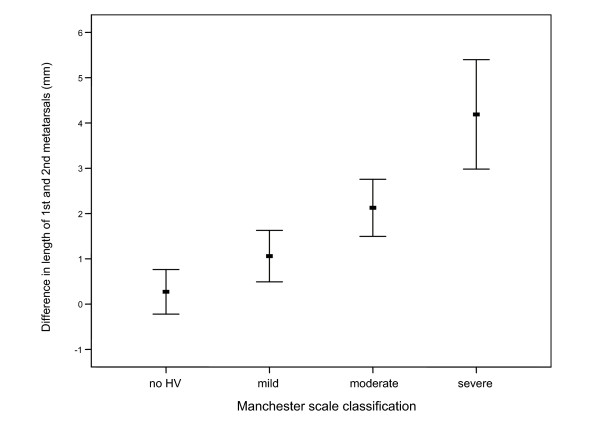
**Mean difference (95% confidence interval) in length of first and second metatarsals between Manchester Scale groups**. Positive values indicate that first metatarsal is longer. Note: no HV = group 1, mild = group 2, moderate = group 3, severe = group 4 on Manchester Scale.

**Figure 3:**
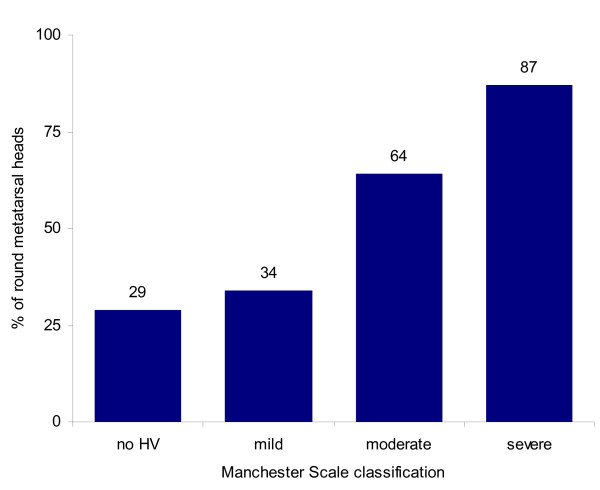
**Percentage of each Manchester Scale group that had a round first metatarsal head**. Note: no HV = group 1, mild = group 2, moderate = group 3, severe = group 4 on Manchester Scale.

When evaluating for differences in x-ray observations between the four Manchester Scale groups, there were a number of significant findings. The mean (or median where appropriate) values of each radiographic observation for the four groups and statistical comparisons are shown in Table 4 (see also Additional File 1 for diagrammatic representation of data). There were two notable findings from the data presented in Table 4, in which a clear relationship can be graphically demonstrated. Firstly, Figure 2 shows the relationship between increasing length of the first metatarsal and increasing severity of hallux valgus. Secondly, Figure 3 demonstrates the increasing proportion of participants with a round first metatarsal head as the severity of hallux valgus increases. Finally, the results also demonstrated that as hallux valgus severity increased, more participants exhibited osteoarthritis of the first metatarsophalangeal joint.

**Table 4:** Comparisons between Manchester Scale groups for radiographic angles and observations (see also Additional File 1 for diagrammatic representation of data).

	Manchester Scale, mean (SD) unless otherwise stated				ANOVA unless otherwise stated	
**Variable**	**0** **(n = 144, 35.8%)**	**1** **(n = 134, 33.3%)**	**2** **(n = 87, 21.7%)**	**3** **(n = 37, 9.2%)**	**F**_**df**_	**p-value**

Hallux abductus angle	11.3 (6.7) ^§^	14.3 (6.7) ^§^	25.4 (7.8) ^§^	37.3 (9.5) ^§^	169.6_3,398_	<0.001
Hallux abductus interphalangeal angle	17.3 (6.9) ^† ‡^	17.1 (7.3) ^# ‡^	14.2 (7.3) ^*^^#^	11.1 (7.7) ^* #^	10.2_3,398_	<0.001
Proximal articular set angle	5.5 (4.1) ^§^	7.6 (5.5) ^§^	13.6 (6.5) ^§^	20.4 (9.2) ^§^	86.1_3,398_	<0.001
Intermetatarsal angle	7.9 (2.1) ^†‡^	8.3 (2.4) ^†‡^	10.6 (3.1) ^§^	13.3 (4.1) ^§^	51.6_3,398_	<0.001
Four grade sesamoid scale - median (range)	0 (0-2)	1 (0-2)	2 (0-3)	3 (1-3)	χ^2^_9df _= 242.1	<0.001
Seven position sesamoid scale - median (range)	2 (1-5)	3 (1-5)	4 (1-7)	6 (3-7)	χ^2^_18df _= 254.5	<0.001
Difference in lengths of first and second metatarsals (+ve indicates longer first metatarsal)	0.3 (3.0) ^†‡^	1.1 (3.3) ^‡^	2.1 (2.3) *^‡^	4.2 (3.6) ^§^	17.6_3,398_	<0.001
Metatarsus adductus angle	18.3 (6.2)	17.3 (6.2)	18.0 (5.5)	19.1 (6.7)	1.2_3,398_	0.314
Simplified metatarsus adductus angle	21.5 (6.9)	21.2 (6.3) ^‡^	22.3 (5.4)	24.5 (8.5) ^#^	2.8_3,398_	0.038
Hallux abductus angle (Miller)	10.6 (6.3) ^§^	13.2 (6.3) ^§^	23.6 (7.6) ^§^	35.6 (9.3) ^§^	169.3_3,398_	<0.001
Distal articular set angle	7.0 (4.1)	7.0 (4.5)	6.4 (5.4)	6.0 (3.9)	1.1_3,398_	0.324
Congruency of first MPJ - median (range)	0 (0-2)	1 (0-3)	1 (0-3)	3 (1-3)	χ^2^_18df _= 266.0	<0.001
First metatarsal declination angle	20.1 (4.0)	19.6 (3.7)	19.6 (3.7)	19.0 (3.5)	0.9_3,398_	0.418
Lateral intermetatarsal angle	2.5 (3.8)	2.5 (3.5)	2.6 (4.0)	1.5 (3.4)	0.7_3,398_	0.577
Calcaneal inclination angle	20.6 (5.4)	19.4 (5.5)	20.4 (5.7)	19.6 (5.4)	1.2_3,398_	0.305
Navicular height/truncated foot length	0.192 (0.038) ^†‡^	0.184 (0.034)	0.176 (0.029) *	0.170 (.038) *	5.7_3,398_	0.001
Osteoarthritis of first MPJ (% OA within group)	44/144 (31%)	51/134 (38%)	48/87 (55%)	30/37 (81%)	χ^2^_3df _= 37.6	<0.001
Round first metatarsal head (% round within group)	42/144 (29%)	46/134 (34%)	56/87 (64%)	32/37 (87%)	χ^2^_3df _= 59.8	<0.001

Note: ^*^significantly different to grade 0, ^#^significantly different to grade 1, ^†^significantly different to grade 2, ^‡^significantly different to grade 3, ^§^significantly different between all groups

## Discussion

The purpose of this study was to determine the association between the clinical appearance of hallux valgus (using the Manchester Scale) to the radiographic observations of hallux valgus in older people. In addition, the study provides additional insight into potential causes of, or contributing factors to, the development of hallux valgus. Unlike previous studies, this study; (i) had a large sample, (ii) included a range of severity of hallux valgus deformity rather than just cases with or without hallux valgus, and (iii) only included older people, in whom the progression of hallux valgus is likely to have largely ceased.

Prior to evaluating the participants' x-rays, intra-tester reliability was assessed and found to be generally good to excellent, with most reliability coefficients above 0.80. The least reliable observation was the hallux interphalangeal angle (ICC 0.66). Once x-ray measurement reliability was established, we were subsequently interested in whether these measurements were associated with hallux valgus severity. As expected, our results demonstrated a significant linear relationship between the hallux abductus angle and hallux valgus severity. The hallux abductus angle was shown to mirror the clinical appearance of hallux valgus, as it progressively increased with the Manchester Scale. The mean hallux abductus angles (using both traditional and Miller's techniques) between all four Manchester Scale grades differed significantly, indicating a clear division between the four groups. Also as expected, the intermetatarsal angle had a significant relationship with hallux valgus, showing a linear increase as the severity of the deformity progressed. Similar results for the two measurements above have previously been identified in a subset of this sample (n = 190) by Menz and Munteanu [27], and in a sample of 176 patients by Pique-Vidal and Vila [38], in which x-ray measurements were correlated with a 100 mm visual analog scale of hallux valgus severity.

Sesamoid displacement has long been associated with hallux valgus [10,39]. The results of both the four grade and seven position sesamoid scales showed a progressive increase in sesamoid displacement as hallux valgus severity increased. Participants with severe hallux valgus were more likely to have a laterally displaced tibial sesamoid relative to the first metatarsal head compared to participants with no or mild hallux valgus. The results indicate that lateral displacement of the sesamoids relative to the first metatarsal head occurs with an increasing hallux valgus deformity. Conversely, the hallux abductus interphalangeal angle was found to decrease as hallux valgus increased. Similar findings were documented by Menz and Munteanu [27] where the authors reported that the hallux abductus interphalangeal angle had a weak negative correlation with the Manchester Scale. The decrease in the hallux abductus interphalangeal angle in more severe hallux valgus deformities is likely due to increased adductory forces from the second toe, however we cannot be certain of this.

A long first metatarsal has been suspected to be a causative factor for hallux valgus [10,39]. As can be appreciated from Figure 2, there was a linear relationship between increasing relative first metatarsal length and increasing hallux valgus deformity. Participants categorised with severe hallux valgus had, on average, a longer first metatarsal (relative to the second metatarsal) of +4.2 mm. In comparison, participants categorised as having no hallux valgus had, on average, a longer first metatarsal of only +0.3 mm. A long first metatarsal and a greater first metatarsal protrusion have been linked to hallux valgus deformity in previous studies [12,22,40]. Importantly, it can be assumed that metatarsal length is not influenced by hallux valgus deformity (i.e. first metatarsal length is fixed in adulthood and is unlikely to increase as a result of hallux valgus). Because our study is the first to investigate older people only, these results may indicate that a longer first metatarsal may be a contributing factor to the development of hallux valgus, and may predispose to more severe hallux valgus deformity. An explanation for this may be that a longer, protruding hallux is more likely to give way to lateral deviation (hallux valgus formation) as a result of compression from footwear [40,41].

A round first metatarsal head has also been suggested to be a contributing factor to the development of hallux valgus [10,39]. As can be identified in Figure 3, there was a linear increase in the percentage of participants with a round first metatarsal head as hallux valgus severity increased. To highlight this increase, 87% of participants categorised with severe hallux valgus had a round first metatarsal head, compared to only 29% of participants categorised with no hallux valgus. Similar results were found in a previous study where 100 out of 110 people with hallux valgus (91%) had a round first metatarsal head, compared to 20 out of 100 people (20%) without hallux valgus [12]. These findings suggest that people with a round first metatarsal head may be more likely to develop hallux valgus. It has been suggested that a round first metatarsal head is less capable of resisting deforming forces from footwear compared to a square metatarsal head [1]. However, it is also possible that bony remodelling of the first metatarsal occurs as hallux valgus progresses, which may result in a more rounded appearance of the metatarsal head.

The link between hallux valgus and osteoarthritis of the first metatarsophalangeal joint was also explored. Our results indicate a linear increase in the percentage of participants with first metatarsophalangeal joint osteoarthritis as hallux valgus severity increased. Only 31% of participants categorised with no hallux valgus had osteoarthritis of the first metatarsophalangeal joint, while 81% of participants categorised with severe hallux valgus had osteoarthritis. These results indicate that as hallux valgus severity increases, the more likely osteoarthritis of the first metatarsophalangeal joint will be present. Whether this is a "local" phenomenon or is related to generalised osteoarthritis is unclear, as previous research has shown that people with hallux valgus are also more likely to have osteoarthritis in other body regions [16].

Similarly, the results revealed a significant association between increasing deviation of the first metatarsophalangeal joint and severity of hallux valgus. As expected, there was a significant increase in dislocations and subluxations in participants categorised with moderate or severe hallux valgus deformities, compared to participants categorised with no or mild hallux valgus. This indicates that as hallux valgus severity increases, the first metatarsophalangeal joint will often become laterally deviated, leading to subluxation, or in more severe cases, dislocation. In contrast to increased first metatarsal length, both osteoarthritis and incongruency of the first metatarsophalangeal joint are not generally considered to be aetiological factors for the development of hallux valgus; rather, they are likely to develop in response to increasing hallux valgus deformity. However, as the inclusion criteria for this study involved participants over 65 years of age, it cannot be assumed that hallux valgus was the sole contributing factor to the development of osteoarthritis and incongruency of the first metatarsophalangeal joint.

Unlike the radiographic observations above, the metatarsus adductus angle showed no significant relationship with hallux valgus severity, while the simplified metatarsus adductus angle demonstrated a significant but poor correlation with hallux valgus severity (Spearman's rho 0.110, p = 0.027). These results differ from previous research in which significant relationships have been found between metatarsus adductus and hallux valgus [42,43]. Furthermore, Ferrari and co-workers [44], who also measured the simplified angle, found a moderate positive correlation between metatarsus adductus and hallux valgus as measured from an x-ray. Metatarsus adductus was present in 55% of participants with hallux valgus compared to only 19% among the control group. However, unlike the current study (mean age of 75.9), participants in Ferrari's study were under forty years of age, which may indicate that metatarsus adductus may be more related to juvenile or early stage hallux valgus deformities, but has a weaker relationship with older onset hallux valgus. Differences between the studies in the strength of association may also be due to Ferrari and co-workers investigating the association between two continuous measures, while we investigated the association between a continuous measure (i.e. metatarsus adductus measured in degrees) and an ordinal measure (i.e. hallux valgus measured by the Manchester Scale). As such, it is not possible to make a direct comparison between our study and theirs.

The relationship between foot posture measurements and hallux valgus was also examined. The results revealed no significant relationship between foot posture measurements and hallux valgus, with the exception of navicular height. Our results demonstrated that navicular height decreases as hallux valgus severity increases, however the correlation was only weak (r = -0.217). This finding indicates that either a lower arch height may be a causative factor for the development of hallux valgus, or alternatively, increasing severity of hallux valgus may cause the height of the arch to decrease. While few studies have investigated the relationship between foot posture and hallux valgus, one study found that people with hallux valgus had a greater single-leg resting calcaneal stance position, increased peak pressure and force-time integral under the hallux, and increased force under the central forefoot compared to people without the condition [2]. Because of the weak relationship between arch height and hallux valgus severity identified in our study, we suggest further research is needed to investigate this relationship more definitively.

The findings of this study need to be considered in light of a few limitations. The key limitation of this study is the use of a case-control study design, which does not allow for temporal relationships between variables to be adequately evaluated. In order to ascertain whether these x-ray observations occurred before, and therefore contributed to, the development of hallux valgus, a prospective cohort study would need to be conducted. However, this would be very difficult to undertake, as hallux valgus deformity may take several decades to develop. In the absence of such a study, evidence from case-control investigations can provide useful insights, provided that the associations between variables are strong, dose-dependent and biologically plausible [45]. In this context, the association between a longer first metatarsal and hallux valgus is more likely to be causal than other associations we identified. Our use of an older sample may also strengthen the case for a causal relationship, as it is unlikely that the severity of hallux valgus would progress much further in this age-group. An additional limitation is that our sample did not include people with hallux valgus that had previously had surgical correction. Accordingly, our sample may have included relatively mild cases of hallux valgus that were in some way different to people that had sought out surgery. However, 114 cases (30.8%) had moderate or severe hallux valgus, so our sample had a reasonable representation of more severe cases.

Despite these limitations, these findings indicate that clinical observation of hallux valgus using the Manchester Scale provides useful insights into the progressive nature of the condition, as evidenced by radiographic observations indicative of structural deformity and joint degeneration. Although many of these structural changes are likely to have developed in response to hallux valgus, it is possible that increased first metatarsal length relative to the second metatarsal is a contributing factor to the development and/or progression of hallux valgus.

## Conclusions

Strong associations are evident between the clinical appearance of hallux valgus across four categories of severity and a number of hallux valgus-related x-ray observations indicative of structural deformity and joint degeneration. These findings highlight the progressive nature of the condition and provide further validation of the Manchester Scale. As it is unlikely that metatarsal length increases as a result of hallux valgus, our observation that the relative length of the first metatarsal increases with hallux valgus severity suggests that this may be a contributing factor to the development and/or progression of hallux valgus.

## Competing interests

HBM, KBL and SEM are Editor-in-Chief, Deputy Editor-in-Chief and Associate Editor, respectively, of *Journal of Foot and Ankle Research*. It is journal policy that editors are removed from the peer review and editorial decision making processes for papers they have co-authored.

## Authors' contributions

HBM conceived the idea for the study. PRD, SEM and GVZ assisted with data collection. PRD and KBL conducted the statistical analysis and drafted the manuscript. All authors contributed to interpretation of the data, and read and approved the final version of the manuscript.

## Supplementary Material

Additional file 1 **Graphical representation of comparisons between Manchester Scale groups for radiographic angles and observations (means and standard deviations shown unless otherwise noted)**. NB: *p < 0.05, **p < 0.01. See Table 4 for tabulated data. Click here for file
