# Supplementary material for: Radiographic correlates of hallux valgus severity in older people
Source: J Foot Ankle Res. 2010 Sep 16;3:20. doi: 10.1186/1757-1146-3-20 (PMC2949715; doi:10.1186/1757-1146-3-20)

Additional data file: Graphical representation of comparisons between Manchester Scale groups for radiographic angles and observations. NB: *$p<0.05$, **$p<0.01$. See Table 4 for tabulated data.

Hallux abductus angle**

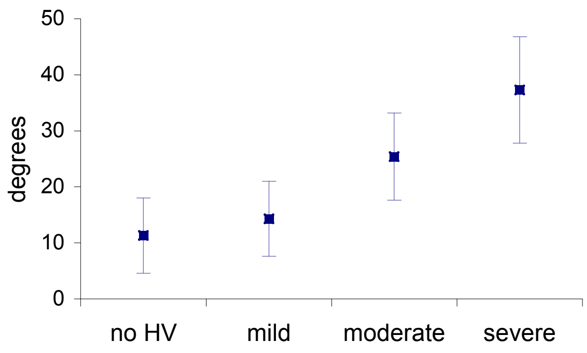

Hallux abductus interphalangeal angle**

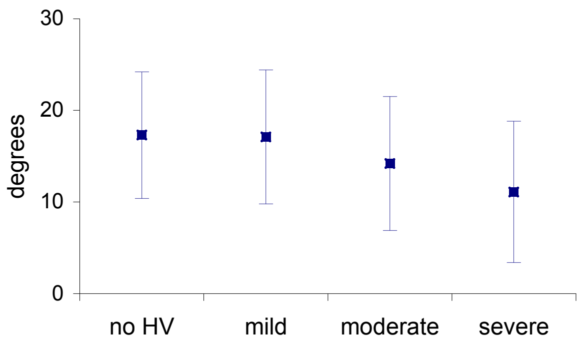

Proximal articular set angle**

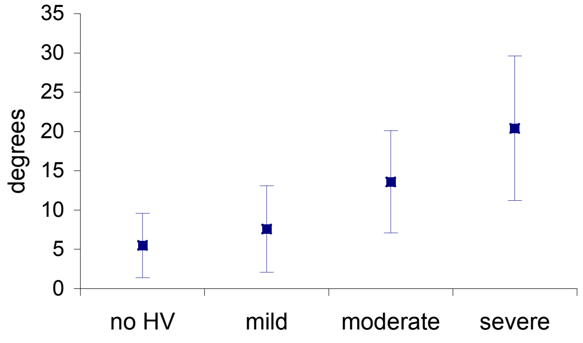

Intermetatarsal angle**

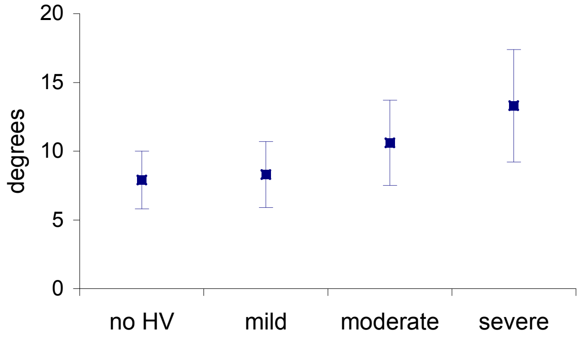

Four grade sesamoid scale**

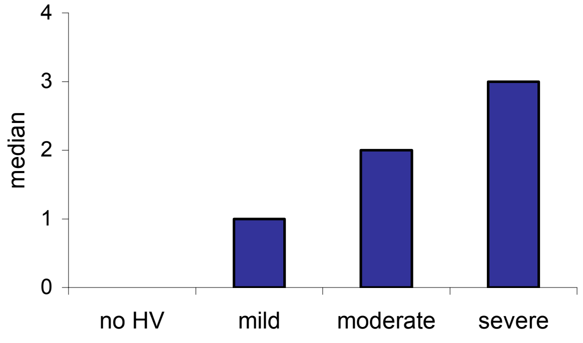

Seven position sesamoid scale**

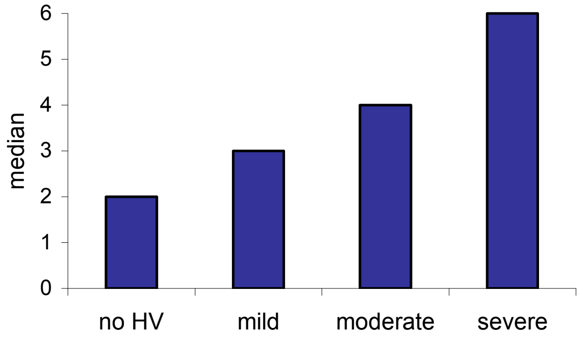

Difference in length of 1st and 2nd metatarsals**

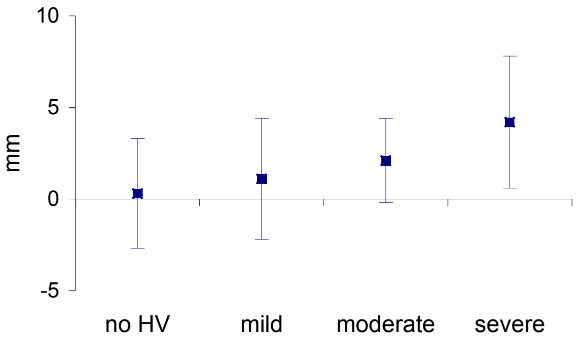

Metatarsus adductus angle

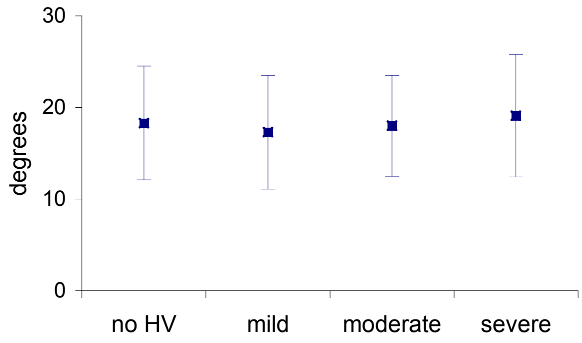

Simplified metatarsus adductus angle*

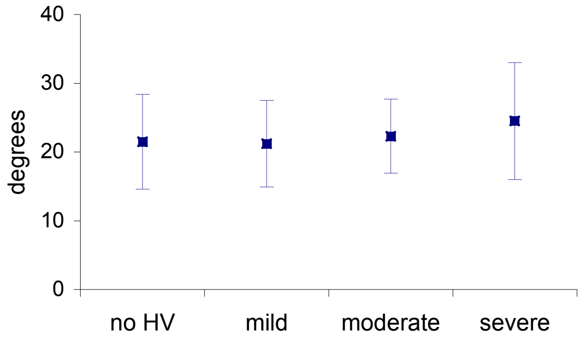

Hallux abductus angle (Miller)**

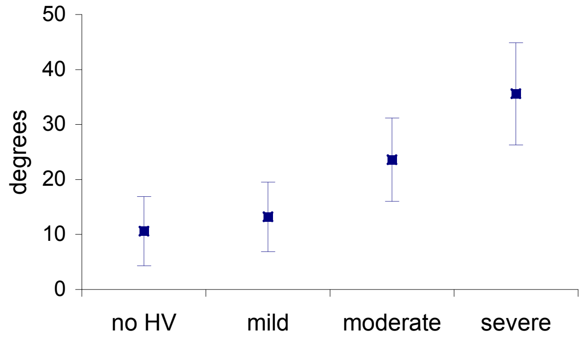

Distal articular set angle

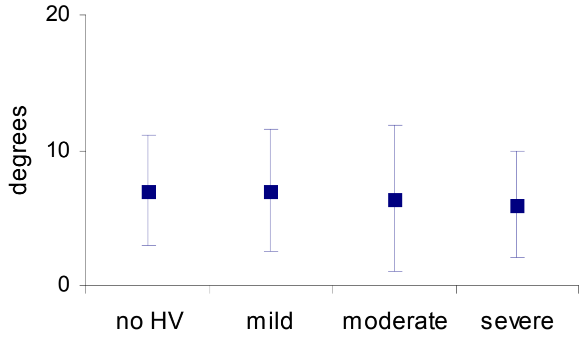

Congruency of 1st MPJ**

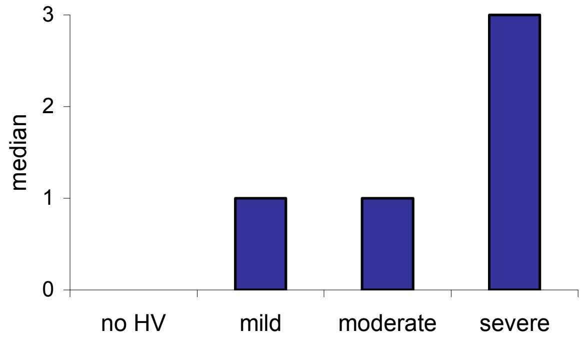

1st metatarsal declination angle

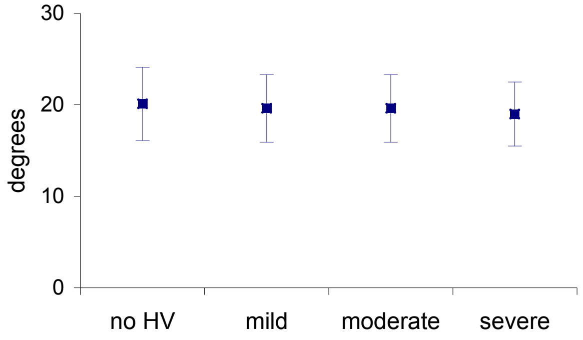

Lateral intermetatarsal angle

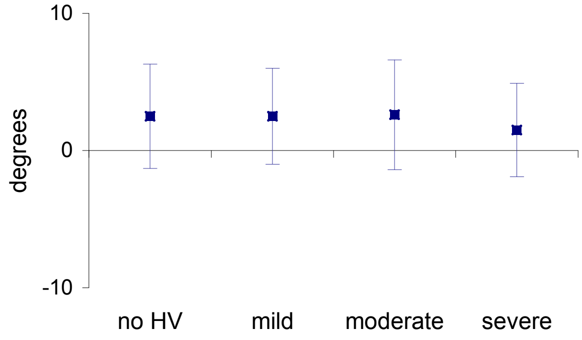

Calcaneal inclination angle

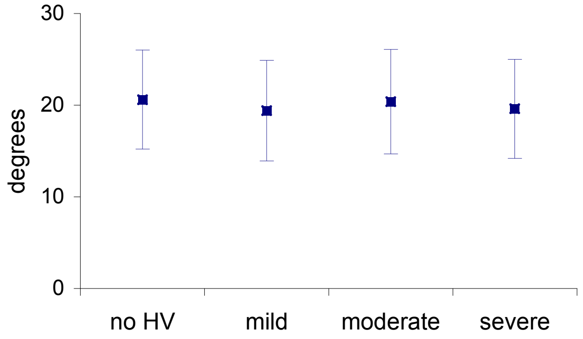

Navicular height / truncated foot length**

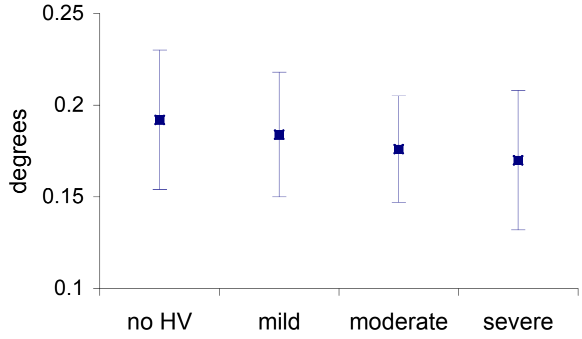

Osteoarthritis of 1st MPJ**

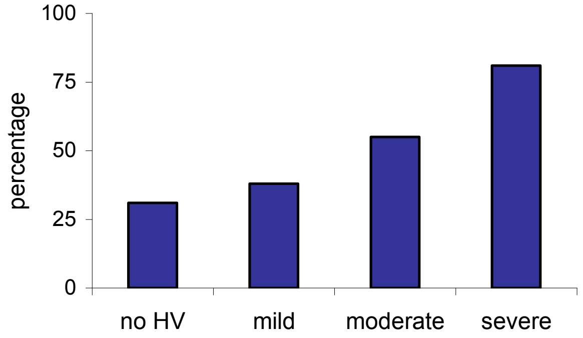

Round 1st metatarsal head**

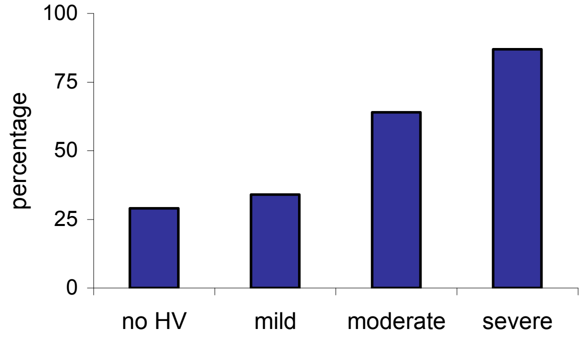

Supplement: Additional file 1 — Graphical representation of comparisons between Manchester Scale groups for radiographic angles and observations (means and standard deviations shown unless otherwise noted). NB: *p < 0.05, **p < 0.01. See Table 4 for tabulated data. [file 1757-1146-3-20-S1.PDF]
